# Exploring the misfolding and self-assembly mechanism of TTR (105–115) peptides by all-atom molecular dynamics simulation

**DOI:** 10.3389/fmolb.2022.982276

**Published:** 2022-08-31

**Authors:** Yuqi Zhang, Yanyan Zhu, Haiyan Yue, Qingjie Zhao, Huiyu Li

**Affiliations:** ^1^ College of Mathematics and Physics, Shanghai University of Electric Power, Shanghai, China; ^2^ Naval Medical Center of PLA, Department of Digestive Diseases, Naval Medical University, Shanghai, China; ^3^ Innovation Research Institute of Traditional Chinese Medicine, Shanghai University of Traditional Chinese Medicine, Shanghai, China

**Keywords:** TTR(105–115), peptide aggregation, *β*-Barrel, hydrophobic interaction, molecular dynamics simulation, ATTR

## Abstract

Pathological aggregation of essentially dissociative Transthyretin (TTR) monomers protein, driven by misfolded and self-interaction, is connected with Amyloid Transthyretin amyloidosis (ATTR) disease. The TTR monomers protein contains several fragments that tend to self-aggregate, such as residue 105–115 sequence [TTR (105–115)]. However, the misfolding and aggregation mechanisms of TTR are still unknown. In this study, we explored the misfolding and self-assembly of TTR (105–115) peptides by all-atom molecular dynamics simulation. Our results indicated that the conformation of the two-peptides appears unstable. In the tetramerization and hexamerization simulations, the results are reversed. When the number of peptides increases, the probability and the length of *β*-Sheet contents increase. Our results show that that the four- and six-peptides both can form *β*-Barrel intermediates and then aggregate into fibers. The critical nucleation for the formation of fibril should be larger than four-peptides. The interactions between hydrophobic residues I107-L111 play an important role in the formation of stable fibrils at an early stage. Our results on the structural ensembles and early aggregation dynamics of TTR (105–115) will be useful to comprehend the nucleation and fibrillization of TTR (105–115).

## Graphical Abstract

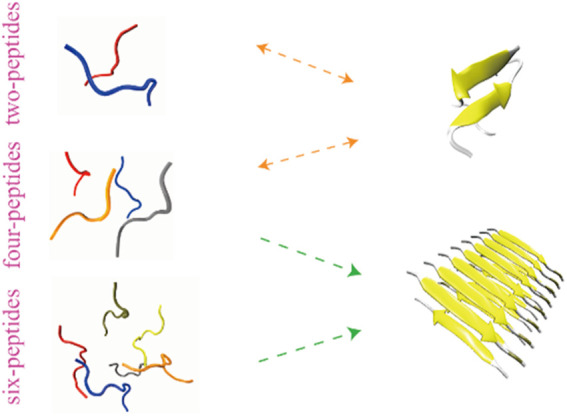

## Introduction

Amyloid Transthyretin amyloidosis (ATTR) is a progressive, fatal disease, including Familial Amyloid Polyneuropathy (FAP), Familial Amyloid Cardiomyopathy (FAC), and Senile Systemic Amyloidosis (SSA). The amyloid deposition of ATTR originated from either wild-type or mutant transthyretin (WT-TTR/M-TTR) causes severe organ damage (Saraiva et al., 1985; Jacobson et al., 1997; Faria et al., 2015). ATTR mostly occurs in western countries, and is also more common in countries such as Portugal, Brazil, Sweden, and Japan etc. (Suhr et al., 2017) Amyloidosis formed by TTR fibrillar aggregates are the primary hallmark of ATTR amyloidosis pathogenesis in the heart or nerve. What’s more, early formed TTR oligomers and protofibrils are neurotoxic (Augustus et al., 2020). TTR, a homotetrameric protein, is composed of four TTR monomers, and one TTR monomer contains 127 amino acids (Hou et al., 2007). TTR tetramer acts as a transporter for thyroid hormone and retinol binding protein in the blood. Generally speaking, TTR is produced from the liver, choroid plexus and retinal, and the native tetramer of TTR mainly exists in plasma (Lorena et al., 2019; Schmidt et al., 2019; Eldhagen et al., 2020). The TTR residues are prone to mutations. So far, more than 130 TTR mutants have been found, most of which are related to amyloid deposition (Gertz et al., 2019).

Nowadays, treatments of ATTR amyloidosis include liver transplantation, Tafamidis and Difunisal etc. (Benson Merrill, 2013; Gertz et al., 2019; Augustus et al., 2020). However, these treatments have certain limitations. For example, liver transplantation is not a treatment for wild type ATTR amyloidosis, but is helpful for hereditary ATTR amyloidosis; Tafamidis and other medicines are used to stabilize the tetramer of TTR. So, it is necessary to explore more drugs that inhibit the formation of ATTR amyloidosis in different stages. Understanding the process of TTR amyloid deposition formation, is essential for exploring therapeutic drugs. In terms of TTR monomers, there are some fragments that are easier to aggregate into fibrous amyloid, such as TTR (105–115) (Jarvis et al., 1994). A TTR monomer is made of eight *β*-Strands (named A through H) and one *α*-Helix, and the peptide TTR (105–115) corresponds to the sequences that are found as *β*-Strands G in the native TTR protein (Saelices et al., 2018). The sequence TTR (105–115) is ^105^Y T I A A L L S P Y S^115^. TTR (105–115) is liable to form protofibrils under acidic conditions (Porrini et al., 2013). Some researchers have explored the conformation character of TTR (105–115) amyloid protofibrils (Lee and Na, 2016) and the binding interactions of TTR (105–115) (Liang et al., 2009). However, the misfolding and aggregation mechanisms of TTR remain unknown. In order to understand the physical mechanism of amyloid deposition and aggregation process, we performed molecular dynamics (MD) simulation method to study the self-assembly process of different numbers on TTR (105–115) peptides. Our research will be conductive to better understand the process resulting in the formation of fibrillization at the first stage, and identify the self-assembly dynamics of TTR peptides. This study will also provide a theoretical basis for the pathogenesis of amyloid deposition-related diseases.

## Materials and methods

### Molecular systems used in simulations

The peptide used in our simulation is taken from Protein Data Bank (PDB) (code:2M5N) and the sequence is ^105^Y T I A A L L S P Y S^115^ (Fitzpatrick Anthony et al., 2013). In order to get initial structure of the TTR (105–115) peptide for the simulations, we performed the MD simulation for 100 ns at 370 K. Then we chose the conformation at 100 nsas the initial structure in Supplementary Figure S1. To understand the contribution of each peptide in the aggregation of TTR (105–115), we systematically performed two-, four-, and six-peptides simulations using all-atom MD simulations. A peptide contains 172 atoms and the total numbers of the atoms are 10,447, 20,269, and 34,482, respectively, for two-, four-, and six-peptides systems. For each research system, seven independent trajectories are obtained starting with different velocities. The initial conformations are shown in Supplementary Figure S1. In oligomerization simulations, the initial configurations with random intermolecular distances and orientations have a minimum intermolecular distance of at least 4 Å. The simulation time of each MD trajectory in all the simulation systems is 1.5 μs. The accumulated simulation time is 31.5 μs The details of all the simulations are summarized in Table 1.

**TABLE 1:** Details of MD simulations of our research systems. Including the size of simulation box, the number of simulated peptides, the time of each MD simulation, the number of MD (run) simulations and the total MD simulation time.

System	Box (nm)	Time (μs)	MD number	Total Time (μs)
Two-peptides	4.757^3^	1.5	7	10.5
Four-peptides	5.873^3^	1.5	7	10.5
Six-peptides	7.060^3^	1.5	7	10.5

### MD simulations

All MD simulations are performed by the all-atom MD simulations with the GROMACS-2018.1 software package (Hess et al., 2008). All systems are performed at 310 K with the AMBER99SB force field (Hornak et al., 2006). Peptides are placed in a cubic box of TIP3P water molecules with a minimum distance to the water box wall of 1.0 nm. The charged residues are not obtained in the TTR (105–115) peptides, and the N- and C-termini are charged (NH3^+^, COO^−^) (Porrini et al., 2013). In order to simulate the human physiological environment, 0.15 mol/L NACL is added to each simulation system. The number of NA^+^/CL^−^ are 10/10, 18/18, and 32/32, for two-, four-, and six-peptides systems, respectively. The visual inspection of three systems is carefully performed using visual molecular dynamics (VMD) (Humphrey et al., 1996).

### Computational analysis methods

We calculated the secondary structure by the Dictionary of Secondary Structure of Protein (DSSP) program (Kabsch and Sander, 1983). If the distance between the atoms (N and O) is within 3.5 Å and the angle (N−H···O) is ≥120° (Zhao et al., 2020), we considered that a hydrogen bond is formed. If the distance between the heavy atoms of two discontinuous residues is ≤5.4 Å, a pairwise residue is considered as contact status. If two or more coherent residues in each chain adopt the *β*-Strand conformation, and at least two backbone hydrogen bonds are obtained among these residues (He et al., 2021), we considered that two chains can form a *β*-Sheet. The cluster conformations are employed the gromos analysis method with a Cα Root Mean Square Deviation (Cα-RMSD) cutoff of 0.4 nm using all the residues. A pairwise residue forming *β*-Sheet is defined as a *β*-Sheet contact (Burkoff Nikolas et al., 2013). A two-dimensional (2D) free energy landscape is constructed using −RTlnH (*x*, *y*), where H (*x*, *y*) is the probability of two selected reaction coordinates, which is the radius gyration (Rg) and *β*-Sheet contact. The Biopython software package is applied to read PDB file (Hamelryck and Manderick, 2003).

## Results and discussion

In order to investigate the dynamics of self-aggregation of TTR (105–115) peptides, we studied three types of polypeptide systems, two-, four-, and six-peptides systems, by all-atom molecular dynamics simulations at normal temperature. To make an effective comparison, the simulation time is 1.5 μs for each simulation. To eliminate the bias of the initial states, the last 400 ns of simulations are selected for analysis.

The convergence of the three systems is checked by comparing all simulations in two different time periods (1,300–1,400 ns and 1,400–1,500 ns). In Supplementary Figure S2, the hydrogen bonds number of TTR (105–115) overlaps well in two time periods of all the simulations, indicating that our three polypeptide systems have the reasonable convergence.

### Secondary structural properties of two-, four-, and six-peptides

**FIGURE 1:**
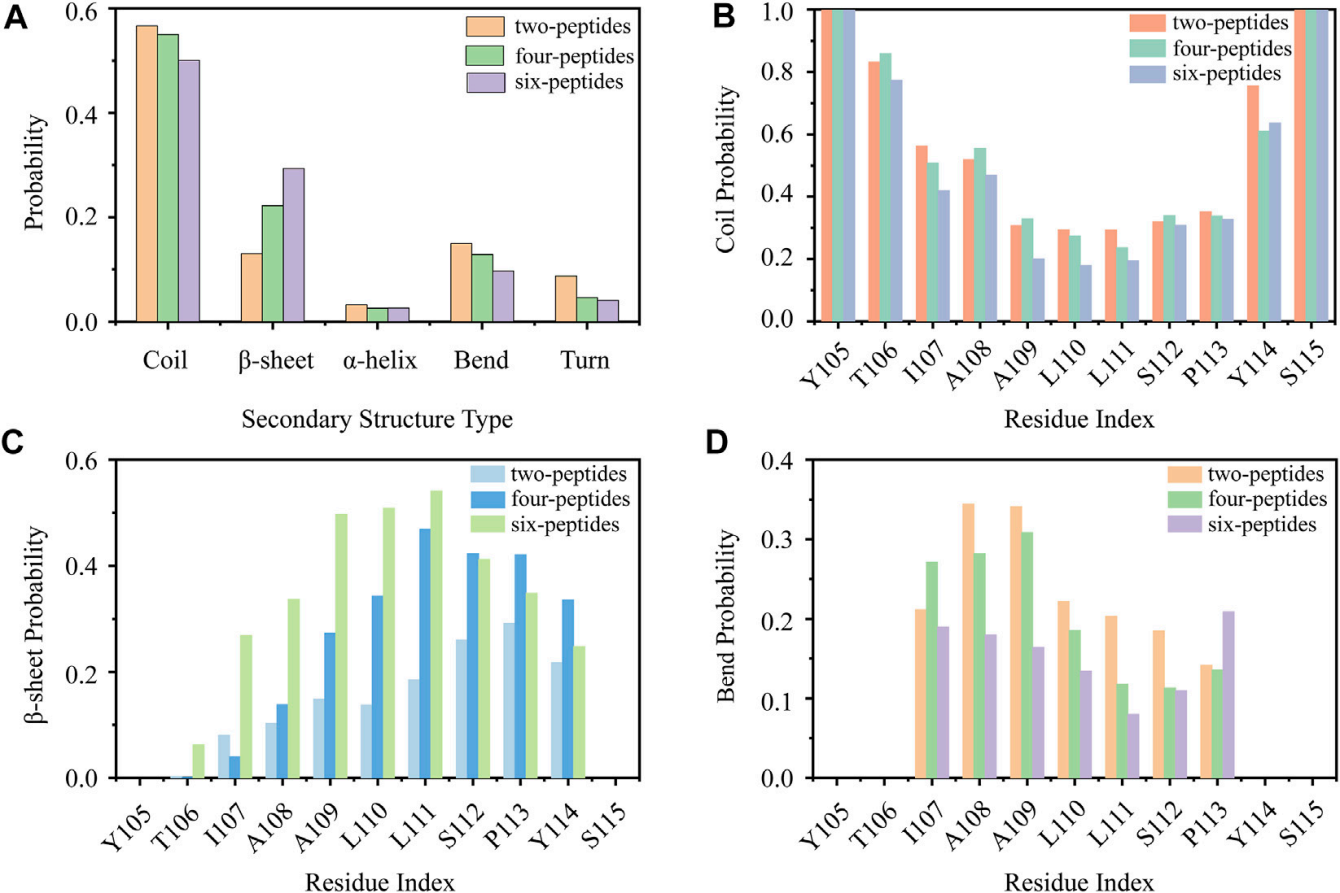
Secondary structure analyses of TTR (105–115). **(A)** The average secondary structure probability contents in terms of coil, *β*-Sheet, *α*-Helix, bend, and turn for TTR (105–115) aggregations with two-, four-, and six-peptides. **(B–D)** The probability of each residue for coil, *β*-Sheet and bend with different TTR (105–115) peptide systems.

First, we analyzed the properties of secondary structures in different systems by discarding the first 1,100 ns data of all the MD runs. As shown in Figure 1A, the average secondary structure properties of each peptide are analyzed in three aggregation systems. For the two-peptides system, the value of random coil reaches ∼57% and the probability of *β*-Sheet is only ∼13%. When the number of peptides reaches four, the probability of random coil decreases to ∼55% and the probability of *β*-Sheet increases to ∼23%. For the six-peptides system, the probabilities of random coil and *β*-Sheet are ∼50% and 31%, respectively. Interestingly, the probability of *α*-Helix is around 3.2%, 2.5%, 2.6% in three systems, respectively. In Figures 1B–D and Supplementary Figure S3, the average secondary structure properties of each residue are analyzed in three aggregation systems. From the figures, we can see that the two terminal residues adopt coil propensity, while the probability of *β*-Sheet increases with the augment of peptides number. Especially, when the number of simulated peptides is six, most residues of TTR (105–115) predominantly form *β*-Sheets (>20%), as shown in Figures 1B,C. And the residue L111 has the highest *β*-Sheet probability of ∼57% in the six-peptides system. For the three aggregation systems, the residues A108 and A109 perform a higher bend conformation (>15%) (Figure 1D) and the residue I107 also has a bend/turn conformation propensity of ∼10%–30% (Figure 1D; Supplementary Figure S3). The probability of *α*-Helix decreases slightly with the increase of the peptides number, as shown in Supplementary Figure S3. The residues A109-P113 display a high tendency to adopt the *α*-Helix conformation. In short, the probability of *β*-Sheet increases with the number of peptides increasing, and the probability of other secondary structures decreases accordingly.

### Conformational differences of two-, four-, and six-peptides of TTR (105–115) system

**FIGURE 2:**
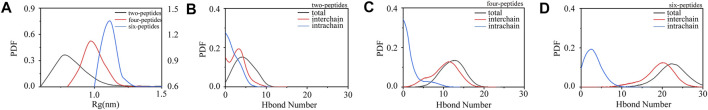
Conformational differences of two-, four-, and six-peptides of TTR (105–115). **(A)** The radius of gyration is presented as function of probability in each research system. **(B–D)** The probability distribution function of the number of intrachain, interchain, and total backbone hydrogen bonds for the two-, four-, and six-peptides systems.

In order to study the aggregation dynamics of different simulated systems, we performed the probability density function (PDF) of Rg and interchain/intrachain hydrogen bonds for the two-, four-, and six-peptides systems in Figure 2. In Figure 2A, it is obvious that the Rg has a peak value around 0.75 nm, and the curve is relatively slow in the two-peptides aggregation system. However, for the four- and six-peptides systems, the peaks of the Rg display around 0.98 and 1.11 nm, respectively. This phenomenon indicated that the structure is more compact in the six-peptides system than that in the two- and four-peptides systems. In Figure 2B, the average values of interchain/intrachain H-bond numbers are 2.935/1.774, respectively, in the two-peptides system. This phenomenon shows that there is no significant difference between interchain and intrachain hydrogen bonds in the two-peptides system. However, as shown in Figures 2C,D, in the four- and six-peptides systems, the average values of interchain hydrogen bonds are much larger than those of intrachain hydrogen bonds in the two-peptides system. The main reason for this phenomenon is that the four- and six-peptides can generate steadier *β*-Sheet conformation. As shown in Supplementary Figure S4, it can be obviously discovered that hydrophobic solvent accessible surface area (SASA) is greater than hydrophilic SASA in the three systems, indicating that the oligomers of TTR (105–115) have strong hydrophobic interactions.

**FIGURE 3:**
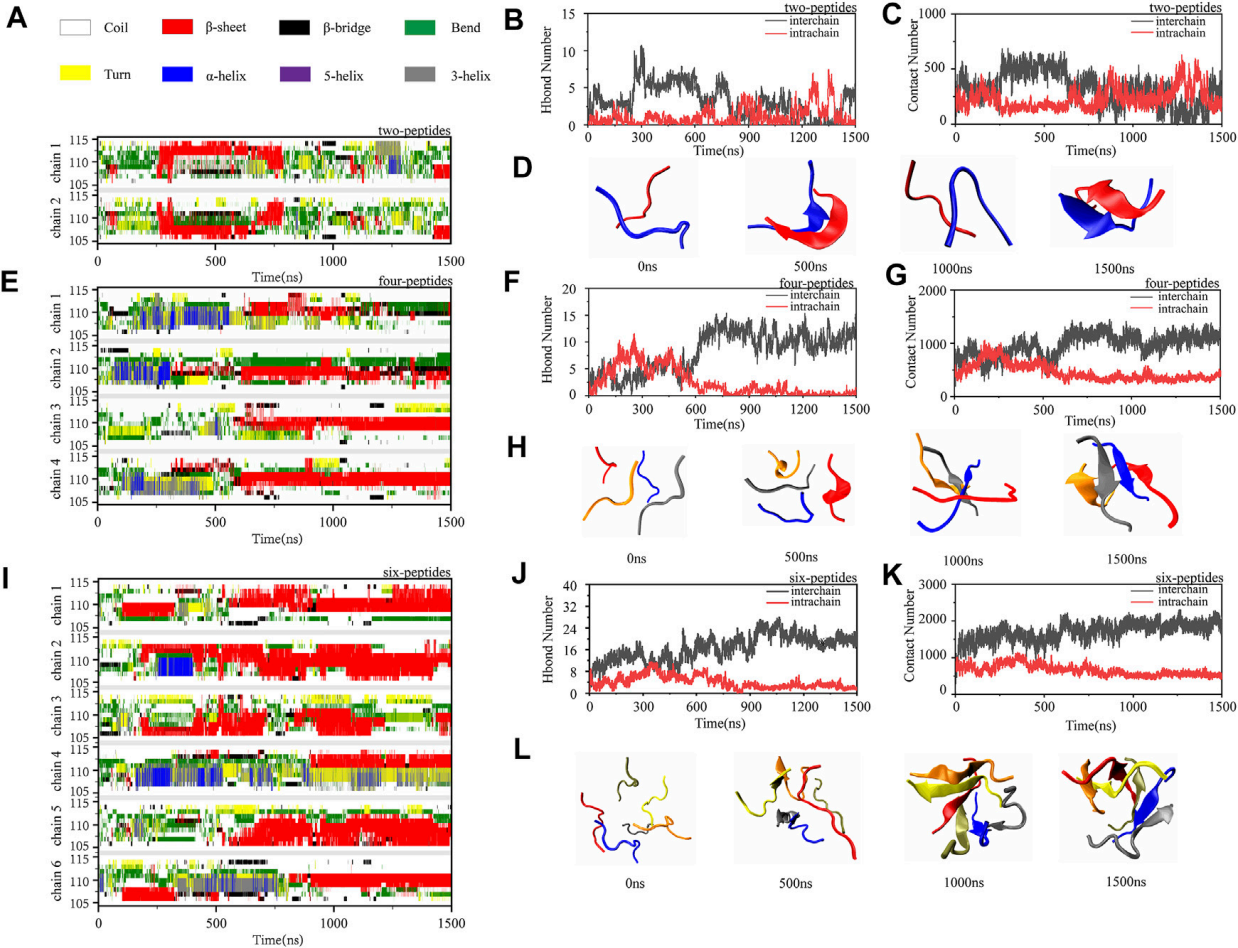
Oligomerization and fibrillization dynamics of TTR (105–115) aggregation. **(A,E,I)** The time evolution of secondary structure for each residue of the two-, four- and six-peptides systems. **(B,C,F,G,J,K)** The number of intrachain and interchain backbone hydrogen bonds and contacts are presented as the function of simulation time for the two-, four-, and six-peptides systems. **(D,H,L)** The structures of two-, four-, and six-peptides were presented.

To investigate the dimerization, tetramerization and hexamerization dynamics of TTR (105–115) aggregation, we showed secondary structure changes, total/interchain/intrachain interactions and snapshots of representative trajectories in Figure 3. As shown in Figure 3A, the representative dimerization trajectory of TTR (105–115) with random conformations shows that it is transient and can be easily converted from random coil into unstructured *β*-Sheet, bend, and turn. Furthermore, the number of residues T106-Y114 adopting *β*-Sheet state is mostly 2–4 in Figure 3A. The intrachain hydrogen bonds and heavy atom contacts also show a slight decline with an augment in *β*-Sheet conformation, as the time evolved (Figures 3B,C). The time evolution of intra- and intermolecular backbone hydrogen bonds correlates to the number of peptides. The representative snapshots every other 500 ns are shown in Figure 3D. During 500 and 1,500 ns, the two-peptides conformations adopt *β*-Sheet contents, and the random coil formations are shown at the other time intervals. For the four-peptides system, the *β*-Sheet contents are relatively stable after 600 ns, and the number of residues T106-Y114 adopting *β*-Sheet state is mostly three to four in Figure 3E. The abundant interchain hydrogen bonds and heavy atom contacts are formed, and the intrachain hydrogen bonds and heavy atom contacts significantly decrease in the four-peptides system (Figures 3F,G). From the representative snapshots shown in Figure 3H for the four-peptides system, we can see that an aggregation progression is performed from random coil to *β*-Sheet-rich contents. For the six-peptides system, it has relatively stable secondary structure and the residues T106∼Y114 adopt *β*-Sheet conformations (Figure 3I). Similar to the four-peptides system, interchain interactions are also dominated in the six-peptides system (Figures 3J,K). As shown in Figure 3L, the representative snapshots are displayed. The six-peptides start from the random coil contents and gradually generate the *β*-Sheet conformation. At 900 ns, the random peptides aggregated into the *β*-Sheet rich contents and the open *β*-Barrel structure can be observed (Figure 3L). The number of residues adopting *β*-Sheet indicates that the *β*-Sheet length is related to the number of peptides. In short, the probability and the length of *β*-Sheet increase with the number of peptides (Scalone et al., 2022), and the probability of other secondary structures decreases accordingly.

To further explore the relationship of the different secondary structures, Supplementary Figure S5 shows the probability values of coil, *β*-Sheet, and *α*-Helix of the three systems as the time evolution. In Figures 3B,F,J and Supplementary Figure S5, the tendency of *β*-Sheet is consistent with that of interchain hydrogen bonds, while *α*-Helix conformational changes correspond to intrachain hydrogen bonds. It is reported that Human islet amyloid polypeptide can form *β*-Sheet through the accumulation of helical oligomers during self-assembly (Sun et al., 2019a). Interestingly, TTR (105–115) segment shares the same conclusion that the accumulation of helix conformation would increase the sheet probability (Supplementary Figure S5). As shown in the Supplementary Figure S5, the *β*-Sheet probability increases as the *α*-Helix conformation decreases. The conformational conversion from helix to *β*-Sheet can be observed in 1,200–1,400 ns for the two-peptides system, 100–500 ns for the four-peptides system, and 300–450 ns for the six-peptides system (Supplementary Figures S5A–C). In addition, in the two-peptides system, the frequent aggregation and dissociation dynamics of *β*-Sheet conformation are undergone, and the dimer adopting *β*-Sheet contents is short-lived (Supplementary Figure S5A). When the peptide numbers are four or six, the *β*-Sheet conformation remains stable (Supplementary Figures S5B,C).

### Self-aggregation free energy landscapes of TTR (105–115)

**FIGURE 4:**
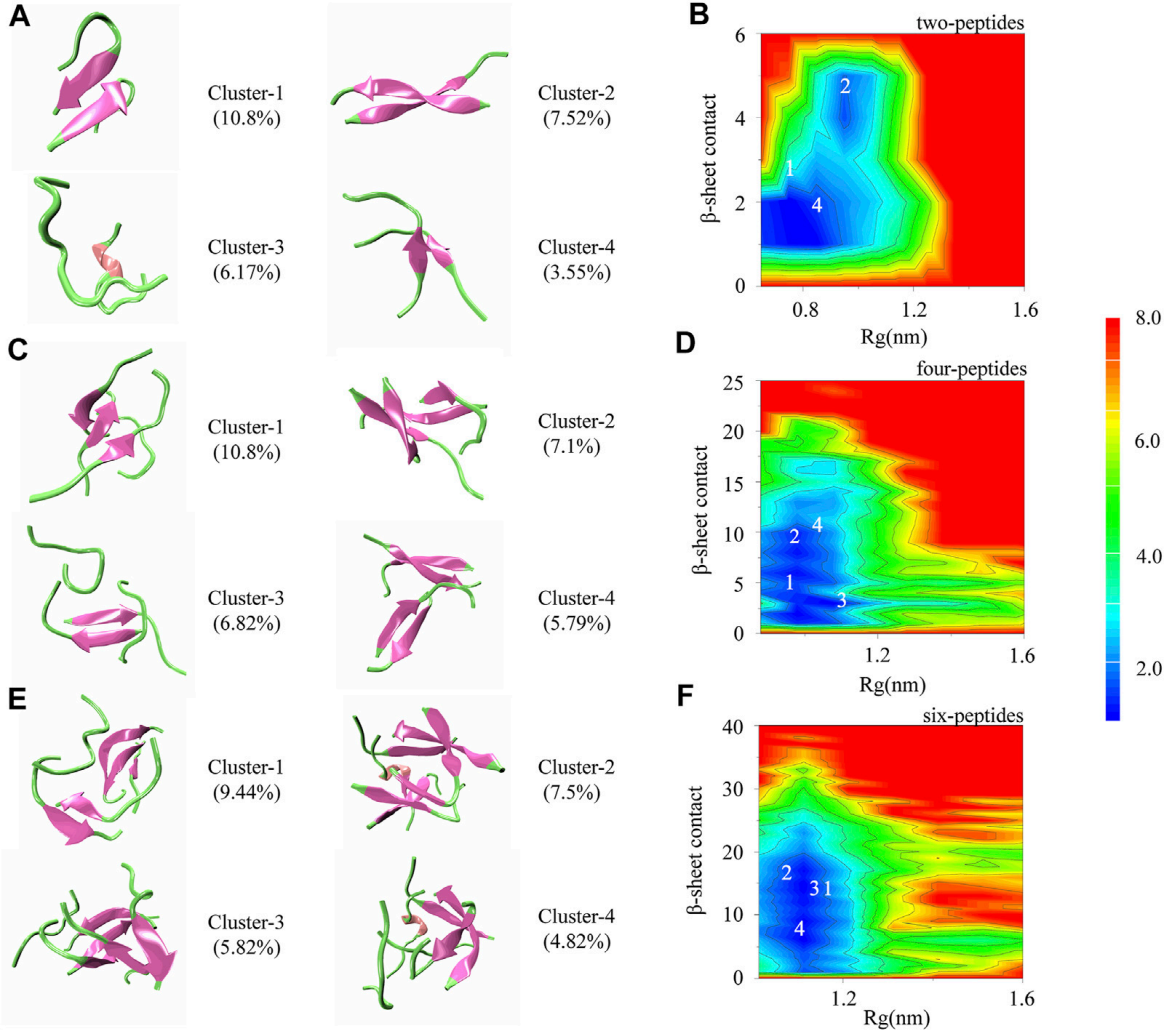
Cluster structure and conformational free energy landscape of TTR (105–115) simulation. Representative conformations of the top four most-populated clusters for two-**(A)**, four-**(C)** and six-**(E)** peptides. **(B,D,F)** The 2D potential mean force (PMF) as a function of radius of gyration (Rg) and *β*-Sheet contact for two-, four-, and six-peptides.

To better understand the self-aggregation dynamics of dimerization, tetramerization and hexamerization of TTR (105–115), we calculated the potential of mean force (PMF) as a function of *β*-Sheet contact and Rg. The clusters analysis for different peptide systems are also shown in Figure 4. In order to eliminate the deviation caused by the initial structures, we conducted the last 400 ns out of 1,500 ns trajectories in the seven independent simulations for the three research systems.

There are 1,456 clusters for the two-peptides system. In Figure 4A, the first, second and fourth clusters, with the populations of 10.8%, 7.52%, and 3.55%, show parallel/antiparallel *β*-Sheet conformations for the two-peptides system. For the third cluster, the disordered coil and helix conformations are shown. In Figure 4B, it is clear that there are two minimum-energy basins, which are approximately at 1–3 (the *β*-Sheet contact) and 0.65–1.0 nm (the value of Rg), as well as 3–5 and 0.9–1.05 nm, respectively. The most representative and popular structures, Cluster-1, -2, and -4, are marked in Figure 4B.

There are 3,93,433 clusters for the four- and six-peptides systems. In Figures 4C,E, the clusters perform abundant conformation of *β*-Sheet. For the four-peptides system, Cluster-2 contains mixed parallel-antiparallel *β*-Sheet with four strands, with a population of 7.1%, and Cluster-4 comprises two orthogonal *β*-Sheet bilayers, with a population of 5.79%. Cluster-1 (10.8%) consists of three-stranded *β*-Sheets with one random coil chain, and Cluster-3 (6.82%) contains two-stranded *β*-Sheets with two random coil chains. The free energy basins are performed in the four-peptides system. *β*-Sheet contact ∼1–14 and Rg ∼0.88–1.18 nm are shown in Figure 4D, respectively. The locations of four representative clusters are shown in Figure 4D. Cluster-1 and -2 conformations are the most common among the four-peptides system. For the six-peptides system, Cluster-3 (5.82%) contains five-stranded open *β* barrel mixed with one random coil chain, and Cluster-1 (9.44%) comprises orthogonal strands, consisting of 3 + 2 *β*-Sheet bilayers. Cluster-2 (7.5%) contains parallel-antiparallel 3 + 3 *β*-Sheet bilayers, and the part of a peptide expresses the *α*-Helix conformation. Cluster-4 is three-strands *β*-Sheets mixed with a *α*-Helix and two random coils chains, with the population of 4.82%. In the six-peptides system, the values of *β*-Sheet contact and Rg are about 1–25 and 1.0–1.2 nm, respectively (Figure 4F). The four most popular cluster conformations are represented in Figure 4F, among which Cluster-1 and Cluster-2 are the most important manifestations in the six-peptides system.

To investigate well-organized aggregation in the four- and six-peptides systems, we further analyzed the probability of rich *β*-Sheet conformation. It has been reported that the amyloid structures of ^105^YTIAAL^110^ and ^106^TIAALLS^112^ contain two different packings and interfaces (Saelices et al., 2018), namely, antiparallel and parallel conformation. Representative TTR (105–115) conformations of bilayer *β*-Sheet and *β*-Barrel are shown in Supplementary Figures S6, S7. In the four-peptides system, the structure of bilayer *β*-Sheet mixed parallel and antiparallel contains 2 + 2 *β*-Sheet bilayer, with the population of 2.04% (Supplementary Figure S6). For *β*-Barrel structure, it includes three- and four-stranded open/closed *β*-Barrels (Supplementary Figure S6). The open-barrel structure with four-stranded *β*-Sheet (β4) is the most popular in all the conformations. In the six-peptides system, the conformations mixed parallel and antiparallel adopt 2 + 2 (0.21%), 3 + 2 (4.3%), 3 + 3 (0.12%) and 4 + 2 (0.2%) *β*-Sheet bilayers (Supplementary Figure S7). The *β*-Barrel structures contain four-, five-, and six-stranded open/closed *β*-Barrels, and open barrel with five-stranded *β*-Sheet possesses the highest probability of occurrence with 5.82% (Supplementary Figure S7). The result shows that as the number of the peptides increases from four to six, the number of strands that can form self-assembly also increases. In other words, if there are six peptides in the system (Supplementary Figure S6), the five- and six-stranded *β*-Sheet conformation will be formed. Combined with the cluster analysis of *β*-Sheet conformation (Supplementary Figures S6, S7), our analyses manifest that the critical nucleation for the formation of fibril should be larger than four peptides. This phenomenon is consistent with previous TTR (105–115) related study (Paci et al., 2004).

### Dimerization, tetramerization, and hexamerization residue-residue interaction analyses

**FIGURE 5:**
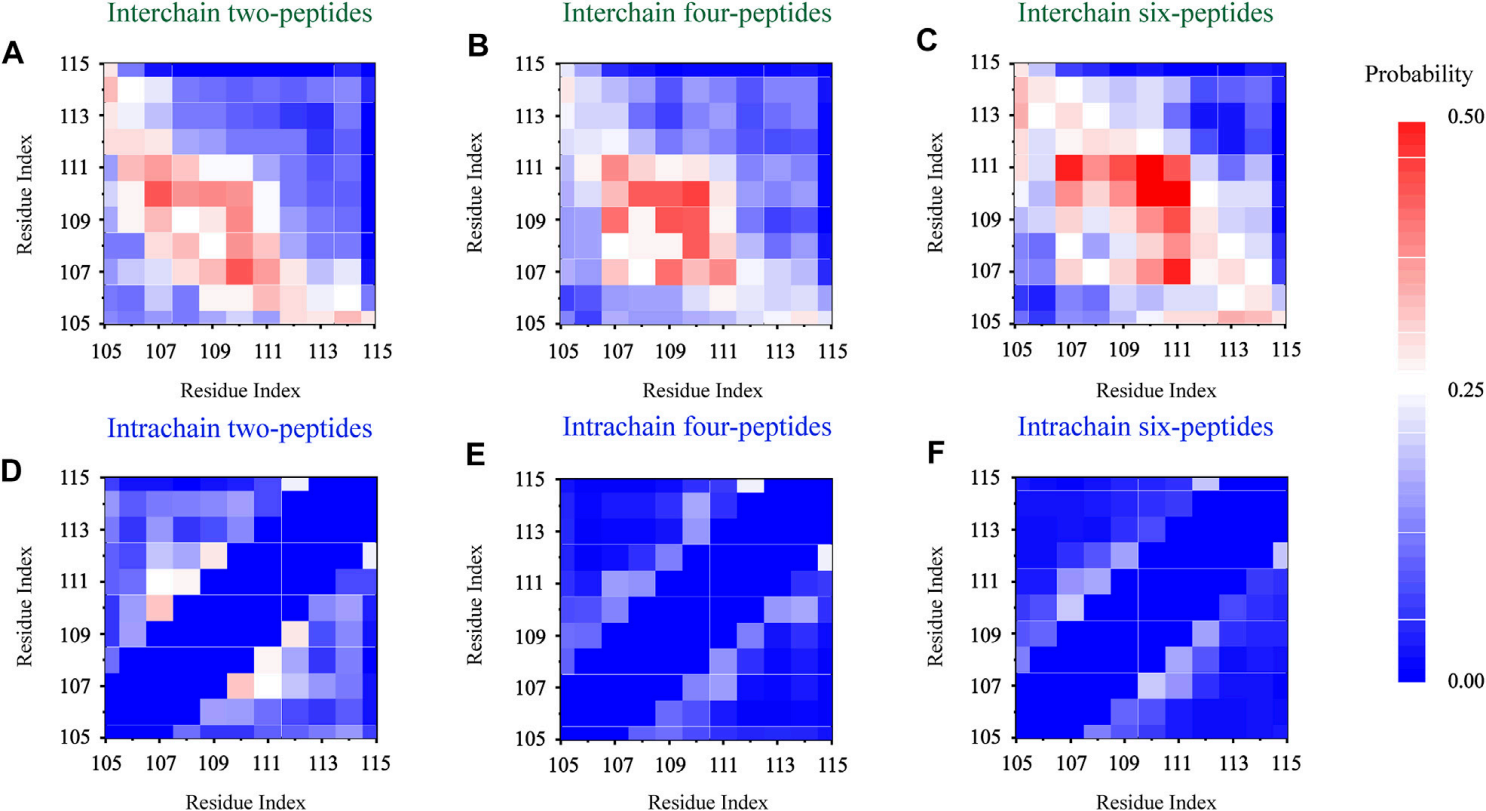
Residue-residue interaction analyses. The interchain and intrachain residue-residue contact probabilities for each simulation of the two- **(A,D)**, four- **(B,E)**, and six- **(C,F)** peptides, respectively.

In order to explore the interaction driving TTR (105–115) aggregation, we calculated the residue pairs contact map of intrachain and interchain for per TTR (105–115) repeat in different systems. In Figure 5A, we can see that in the two-peptides system, the sequence of residues T106-A109 mainly forms the interchain contact, by the formation of parallel or antiparallel *β*-Sheet with the residues A109-S112 in the other chain. It can also be seen in Figures 5A–C that, as the number of peptides increases, interchain interactions between residue pairs increase, and the most popular interchain interactions between residue pairs (L110/I107 for the two-peptides system, L110/L110 for the four-peptides system and L110/L111 for the six-peptides system) also are different. This phenomenon also indicates that, by forming parallel and antiparallel analogous in-register steric zippers, the peptides generate the more stable interaction characteristic, corresponding to Figure 3L. In Figures 5A–C, the interaction of residue pairs is mainly between hydrophobic residues I107-L111, indicating that the hydrophobic residues for interchain interaction play an important role in the research system. What’s more, the hydrophobic residues I107-L111 can interact with interchain residues, and then induce interpeptides to form larger *β*-Sheet size. In the *β*-Sheet conformation of three systems, the *β*-Barrel and bilayer *β*-Sheet of mixed parallel-antiparallel morphology are found in the four- and six-peptides systems (Supplementary Figures S6, S7), and there are both parallel and antiparallel structures in the two-peptides system (Figures 3D, 5A). In addition, the hydrogen bond of the interchain plays an important role in maintaining the stability of the system (Figure 2), especially the four and six-peptides systems, and the important role of the hydrogen bond has also been reported in the other research (Dirix et al., 2005; Liang et al., 2009; Meersman et al., 2011). Our result indicated that the hydrophobic interaction is the principal factor in the formation of stable fibrils at an early stage (Dirix et al., 2005).

In Figure 5D, the intrachain interaction is consistent with the partial helical conformation, such as, *α*-Helix contents in Figure 3A. In Figure 5D, the weak conformation of *β*-Hairpin pattern is consistent with the intrachain interaction around residues T106-A108 and S112-Y114 in the two-peptides system. When the number of peptides increases from 2 to 6, the intrachain interaction between residue pairs transitions from 0.25 (white) to 0 (blue) in Figures 5D–F. The phenomenon indicates the conformational probability of helical and intrachain *β*-Hairpin formations decreases in the four- and six-peptides systems. This result corresponds to the secondary structure in Figures 1, 3. In short, the *β*-Sheet contents of four- and six-peptides are larger than those of two-peptides.

### Two-peptides cannot aggregate into fibers, but four- and six-peptides both can form *β*-Barrel intermediates and then aggregate into fibers

**FIGURE 6:**
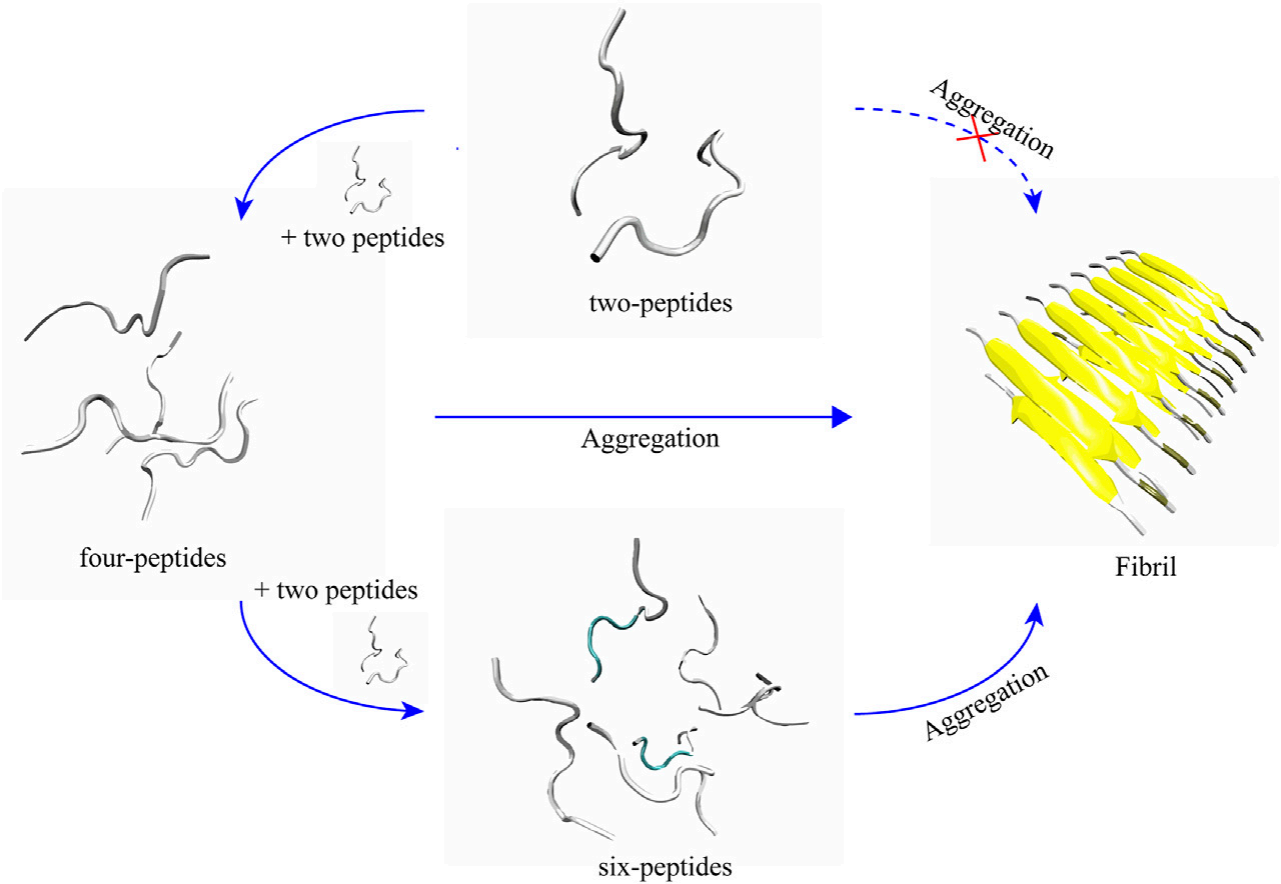
Proposed aggregation mechanism of TTR (105–115) nucleation, based on simulation results. The proposed process of the conformational change and final structural states of two-, four-, and six-peptides are shown.

According to the trajectories of all the simulation systems discussed above, structural state changes of four- and six-peptides are proposed in Figure 6. We found that the structure of the two-peptides is unstable, and the *β*-Sheet can transform into random coil. In other words, the structure of the two-peptides is dynamic and can transfer frequently. For the four-peptides, starting from the coil state, it can transfer into the form of *β*-Barrel or bilayer *β*-Sheet, which is relatively stable. As shown in Supplementary Figure S8A, the four-peptides system can also form a pair *β*-Sheets structure, which will directly transform into the form of random coil. In Supplementary Figure S8B, the four-peptides system can also form two pairs *β*-Sheets structure, which can convert to random coil. As shown in Figure 6, six-peptides can spontaneously form the stable *β*-Barrel or bilayer *β*-Sheet structures. Representative TTR (105–115) conformations of bilayer *β*-Sheet and *β*-Barrel from the cluster analysis of MD simulation for the four- (Supplementary Figure S6) and six-peptides (Supplementary Figure S7) system, show that the total probability of *β*-Barrels is larger than that of the bilayer *β*-Sheet. We proposed that *β*-Barrel could eventually transfer into fibrils. Our results also suggested that the *β*-Barrel might be the potential toxic oligomer of TTR (105–115). The computational and experimental studies also reported that other amyloid fragments (Irbäck and Mitternacht, 2008; Laganowsky et al., 2012; Xie et al., 2013; Do et al., 2016; Zou et al., 2016; Ge et al., 2018; Sun et al., 2018; Sun et al., 2019a; Sun et al., 2019b) such as Aβ_16-22_, hIAPP_8-20_, αS_68-78_, hIAPP_22-29_, Aβ_25-35_, α BC_90-100_, and full-length proteins (Serra-Batiste et al., 2016; Nguyen et al., 2019a; Nguyen et al., 2019b; Sun et al., 2019c; Österlund et al., 2019) such as Aβ, hIAPP, all could form such *β*-Barrels.

The relationship between the propensity of forming *β*-Barrel intermediates and amyloid cytotoxicity was also supported by hIAPP_19-29_ and SOD1_28-38_ assemblies (Sun et al., 2021). We hypothesize that the *β*-Barrel may play a key important role in the amyloid aggregation of TTR (105–115).

## Conclusion

The conformational dynamics mechanism of dimerization, tetramerization, and hexamerization are systematically investigated for TTR (105–115) repeats by all-atom MD simulations with long timescale, accumulatively 31.5 μs for simulation. Our results showed that all the two-peptides structures are very flexible. This structure of two-peptides cannot assemble into fibrils. In the hexamerization simulations, the six-peptides displays the high amyloid aggregation propensities with stable conformation, which contains rich open *β*-Barrel and bilayer *β*-Sheet. In the tetramerization trajectories, they can form both dynamic dimers and rich *β*-Sheet conformations. Hydrophobic residues I107-L111 readily assemble with other chain into intermolecular *β*-Sheet, then the conformation induces other chains to form larger *β*-Sheet size and increase the length of *β*-Sheet. What’s more, the hydrophobic interaction is the main factor in the formation of stable fibrils at an early stage. We also found that the four- and six-peptides both can form *β*-Barrel intermediates and then aggregate into fibers. Our research also suggested that the minimum nucleus unit is not four-peptides, but should be larger than four-peptides. Our study provided an insight of dimerization, tetramerization and hexamerization dynamics mechanism for TTR (105–115) repeats, which will be useful to further research the pathology of TTR aggregation.

## Data Availability

The raw data supporting the conclusion of this article will be made available by the authors, without undue reservation.
