# Supplementary figures and images for: Exploring the misfolding and self-assembly mechanism of TTR (105–115) peptides by all-atom molecular dynamics simulation
Source: Front Mol Biosci. 2022 Aug 31;9:982276. doi: 10.3389/fmolb.2022.982276 (PMC9473747; doi:10.3389/fmolb.2022.982276)

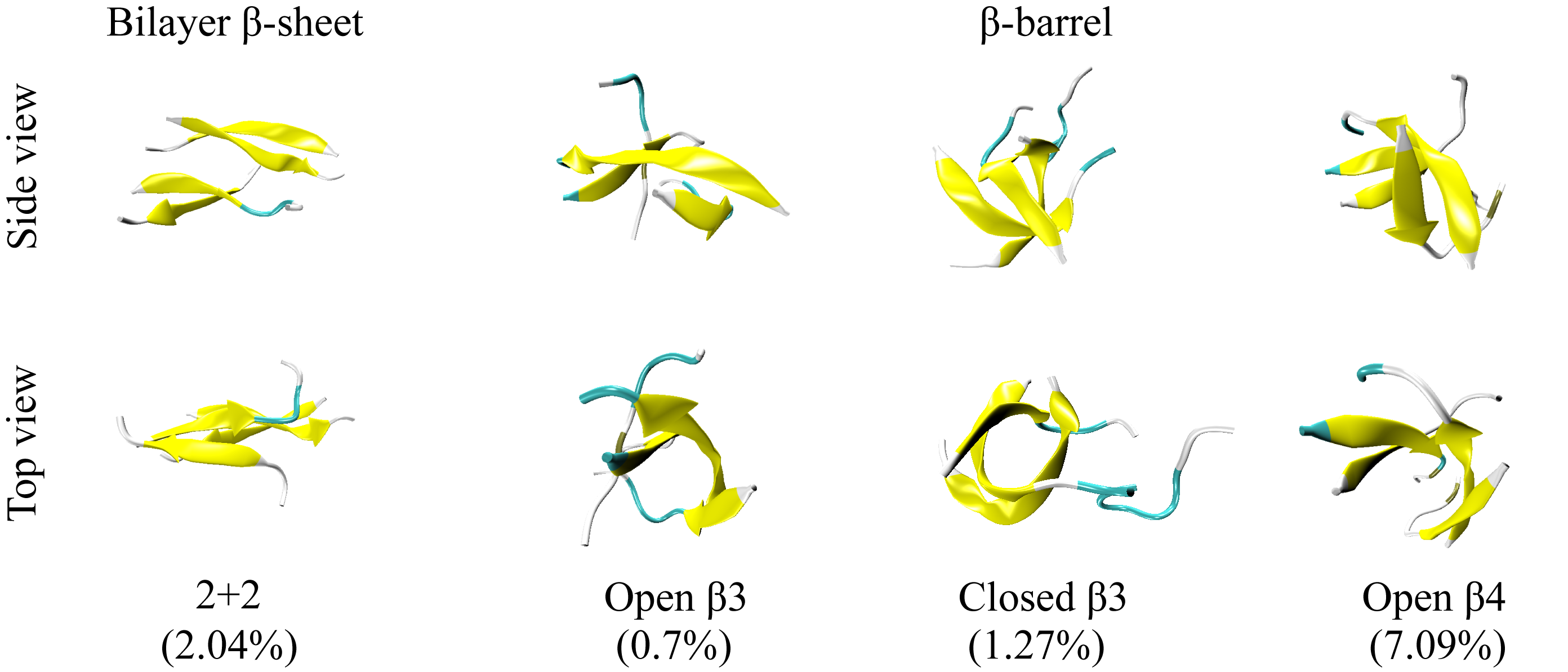

Supplement: Supplementary file 1 [file Image6.TIF]

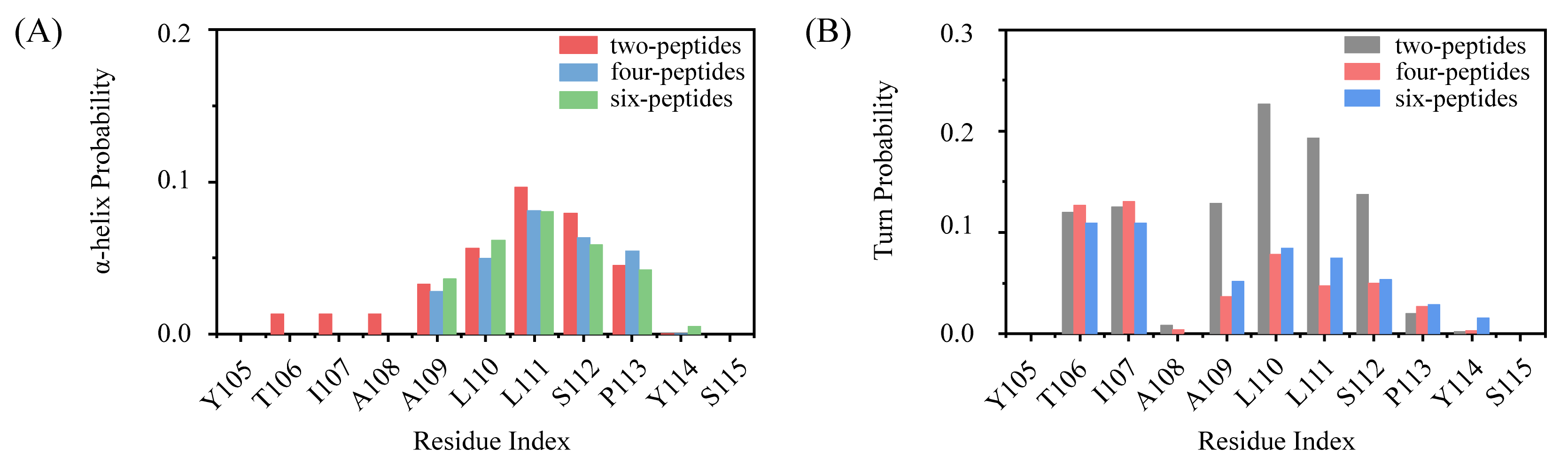

Supplement: Supplementary file 2 [file Image3.TIF]

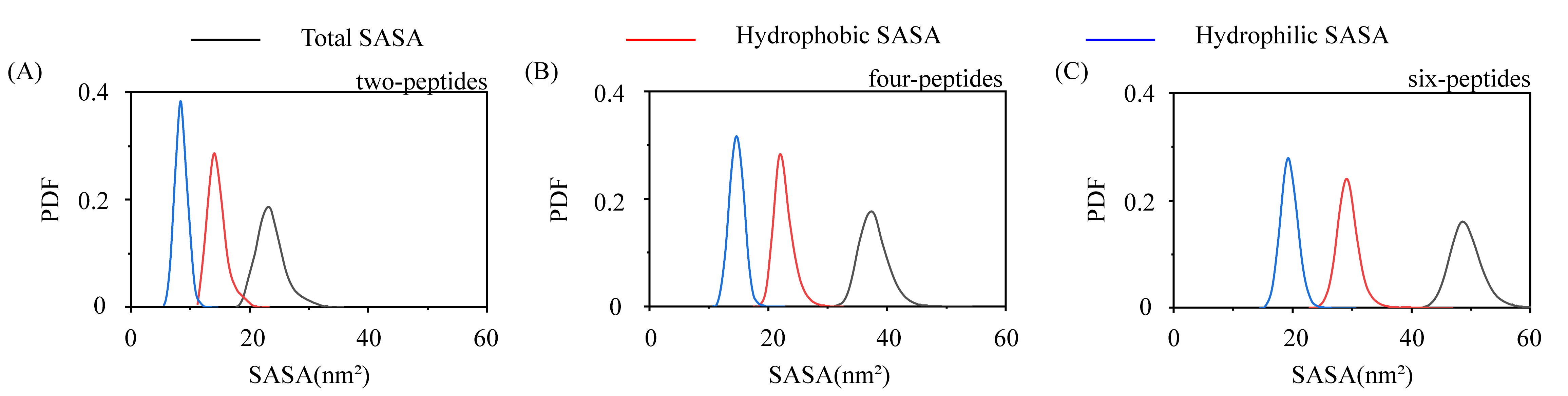

Supplement: Supplementary file 3 [file Image4.TIF]

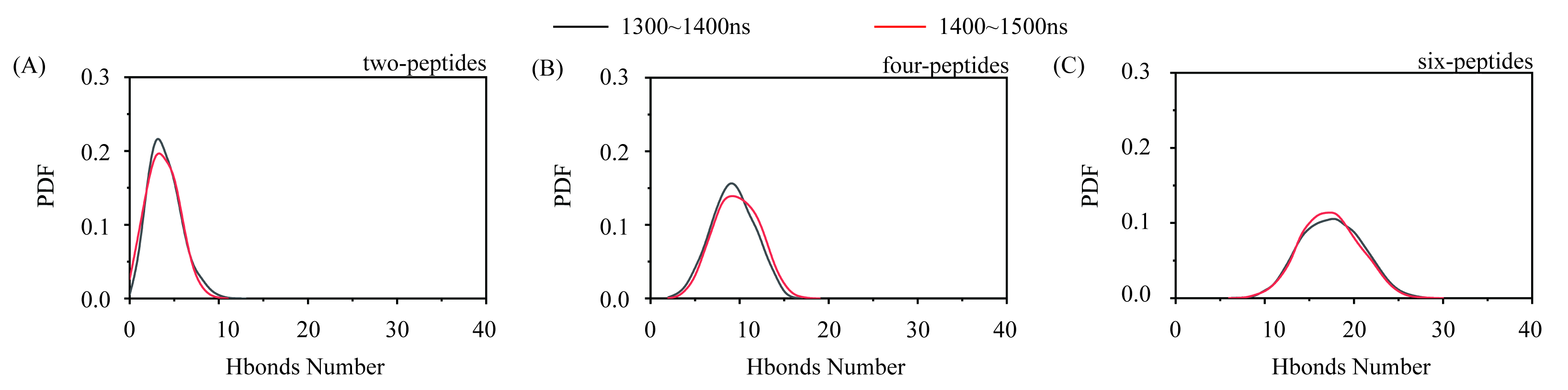

Supplement: Supplementary file 4 [file Image2.TIF]

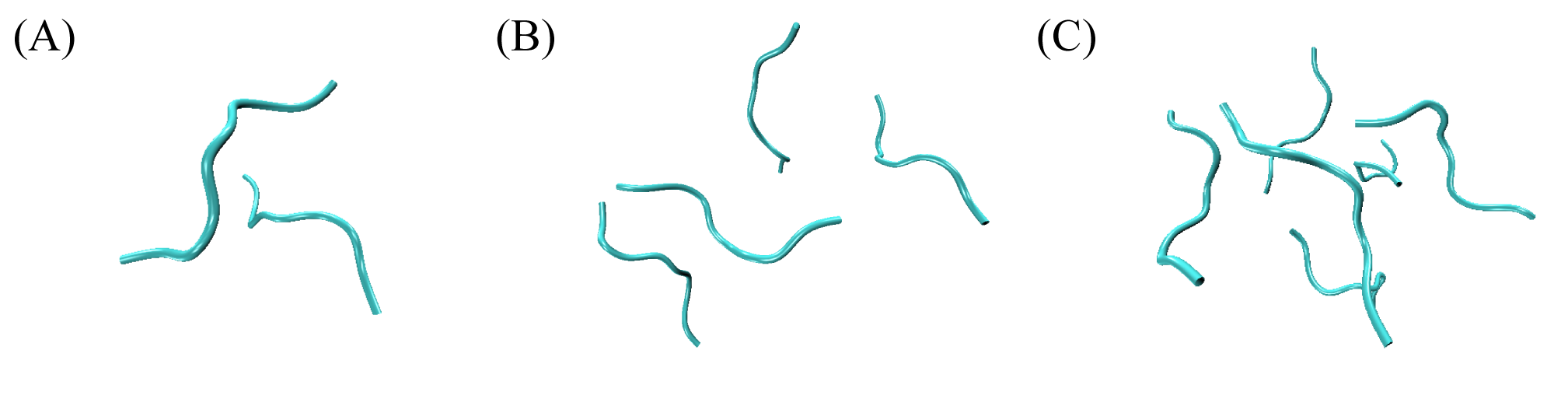

Supplement: Supplementary file 5 [file Image1.TIF]

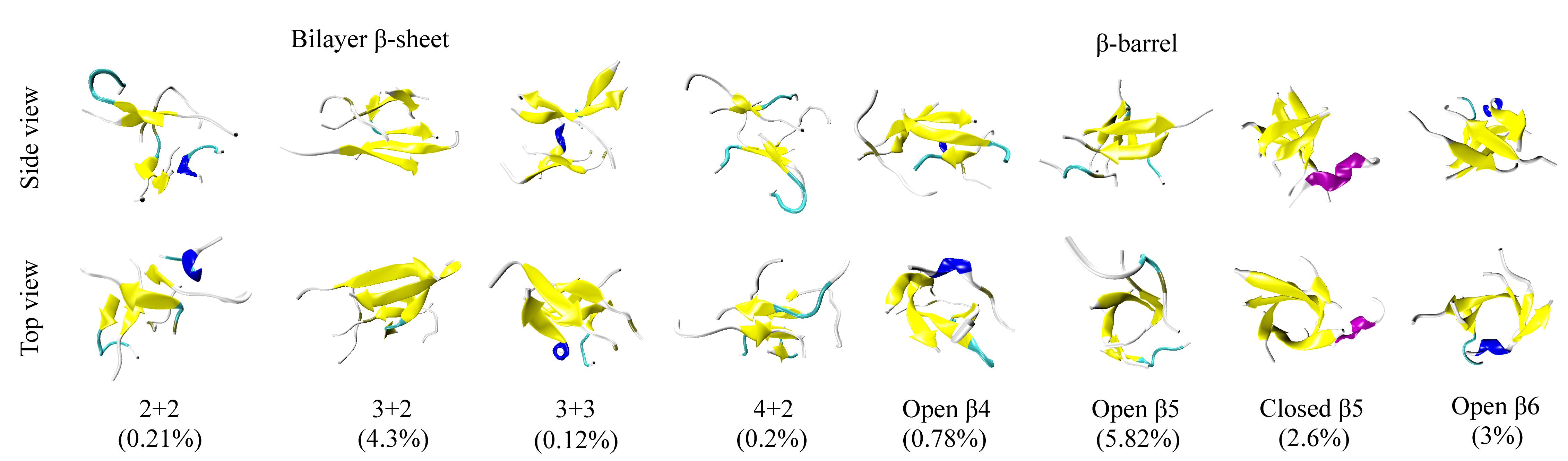

Supplement: Supplementary file 6 [file Image7.TIF]

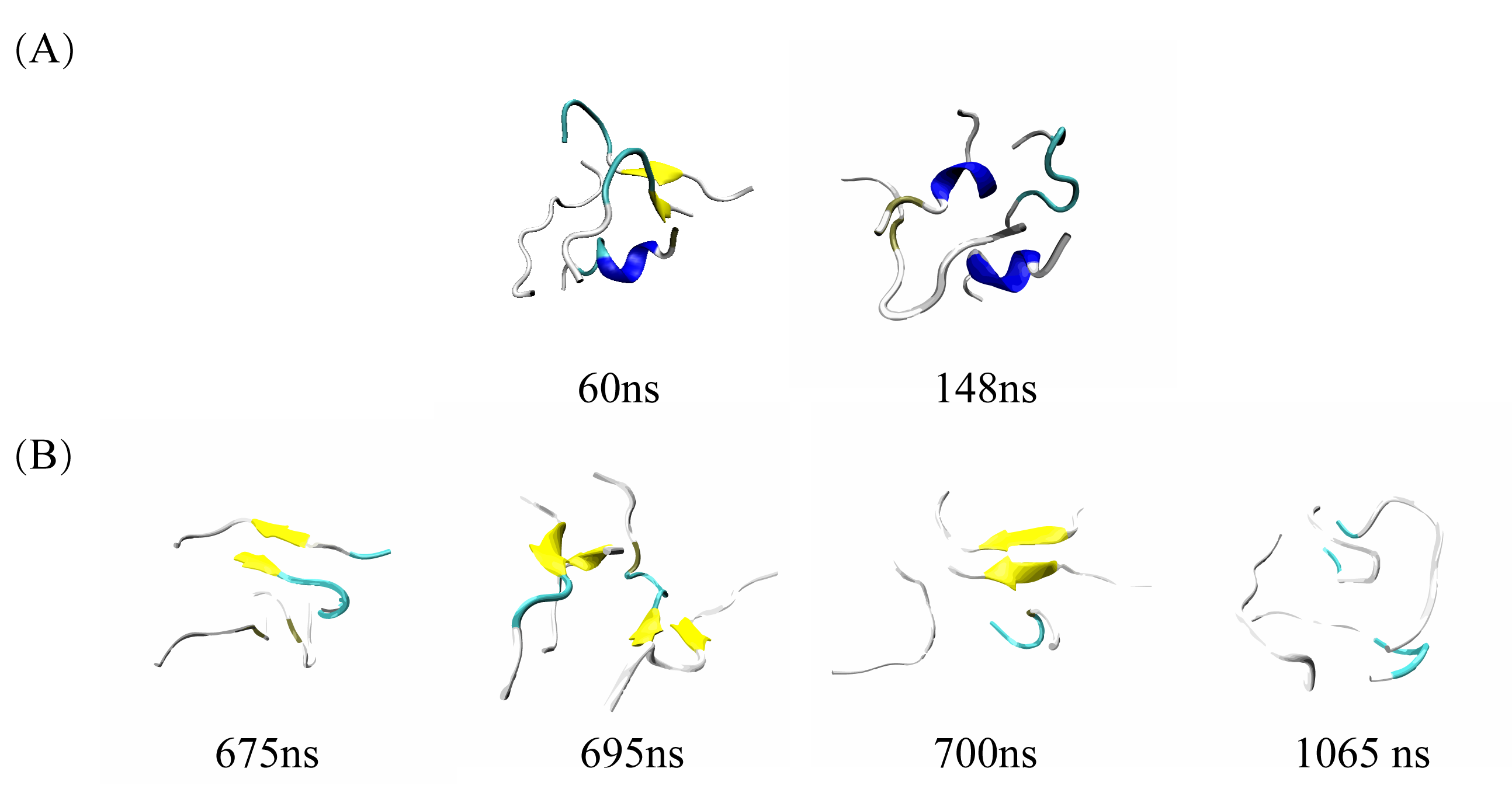

Supplement: Supplementary file 8 [file Image8.TIF]

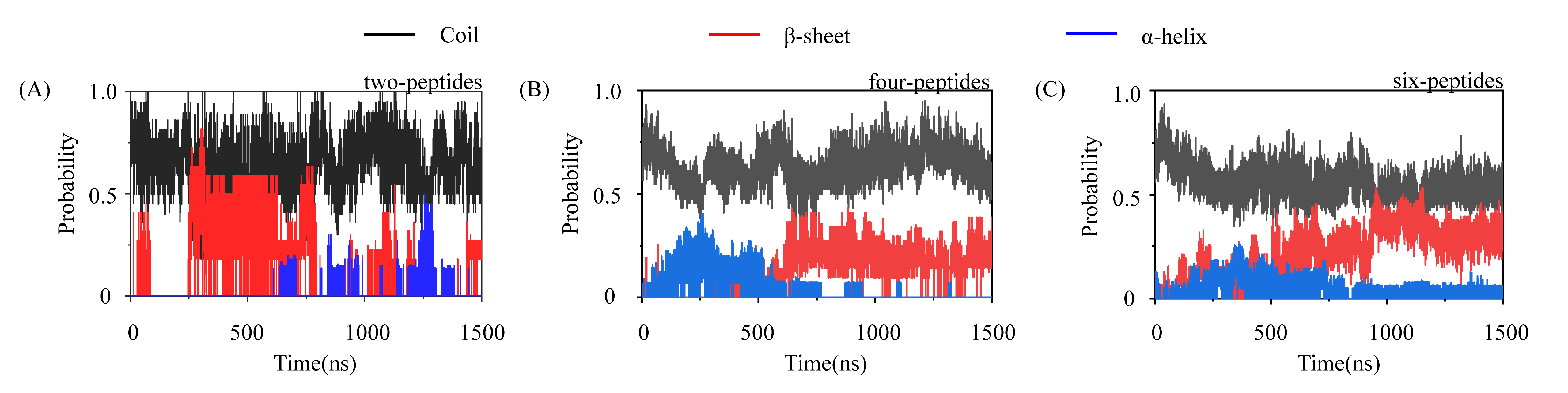

Supplement: Supplementary file 9 [file Image5.TIF]
