# Supplementary material for: Exploring the misfolding and self-assembly mechanism of TTR (105–115) peptides by all-atom molecular dynamics simulation
Source: Front Mol Biosci. 2022 Aug 31;9:982276. doi: 10.3389/fmolb.2022.982276 (PMC9473747; doi:10.3389/fmolb.2022.982276)
Supplement: Supplementary file 7 [file DataSheet1.pdf]

Supplementary Material

1 Supplementary Figures

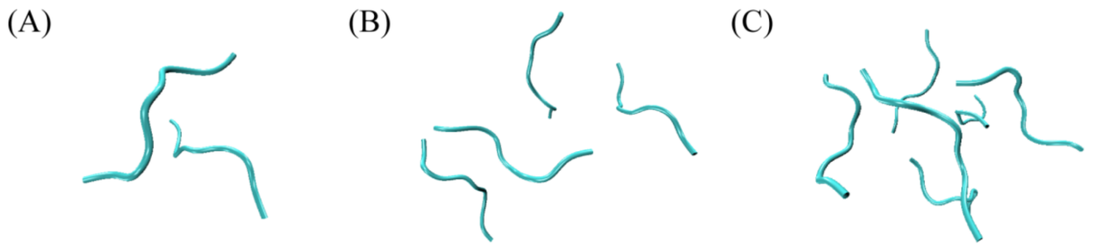

Figure S1. The initial conformations for (A) two-, (B) four- and (C) six- peptide systems.

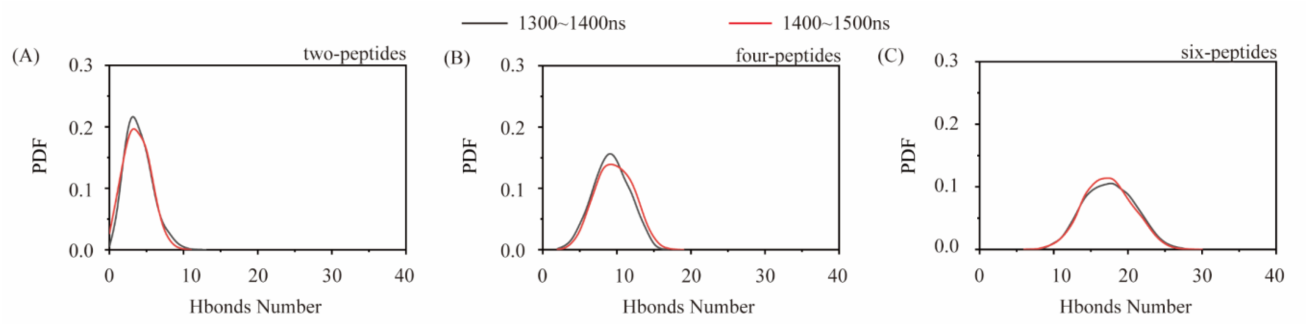

Figure S2. Convergence check of the TTR (105-115) simulations for the (A) two-peptides system, (B) four-peptides system and (C) six-peptides system.

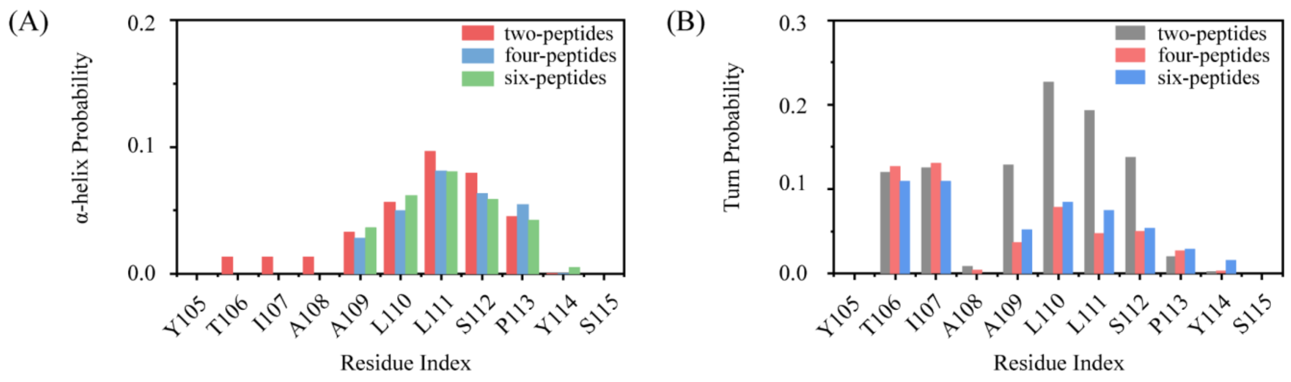

Figure S3. The probability of each residue for α-helix and turn with different TTR (105-115) peptide systems.

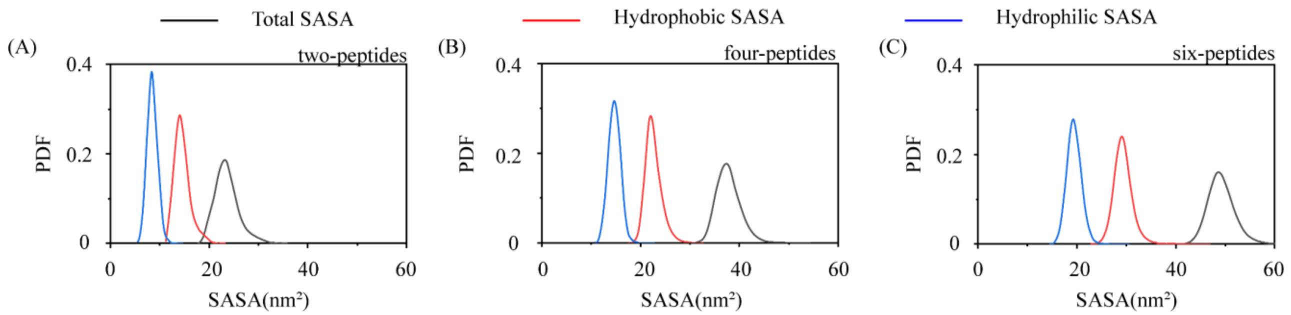

Figure S4. The probability density function (PDF) of the total solvent accessible surface area (SASA), the hydrophobic SASA and the hydrophilic SASA for the (A) two-peptides system, (B) four-peptides system and (C) six-peptides system.

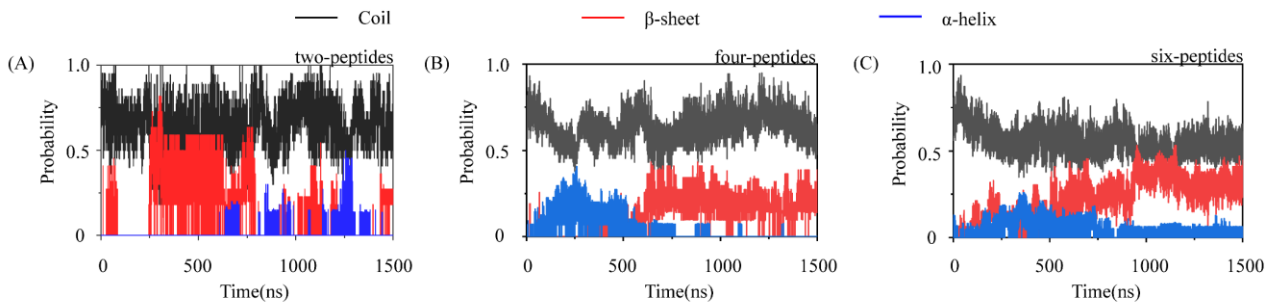

Figure S5. The probability of coil, β-sheet and α-helix as a function of times for the (A) two-peptides system, (B) four-peptides system and (C) six-peptides system.

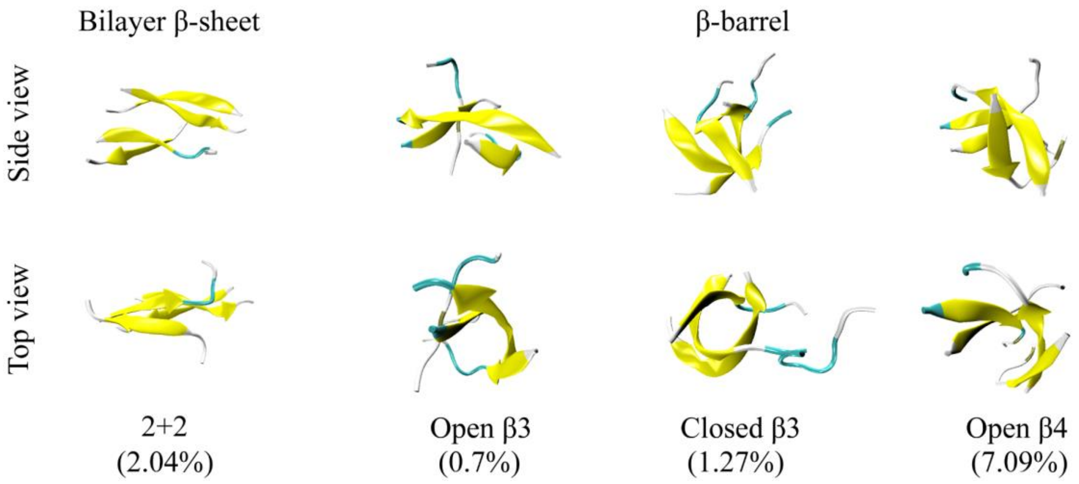

Figure S6. Representative TTR (105-115) conformations of bilayer β-sheet and β-barrel from the cluster analysis of MD simulation for the four-peptides system. Side view and top view were shown. The size of bilayer β-sheet were represented *i*+*j*, where *i* and *j* showed the *i* and *j* stranded β-sheet in each layer, respectively. the n of βn represented n stranded β-barrel, and the type of β-barrel was classed by open and closed.

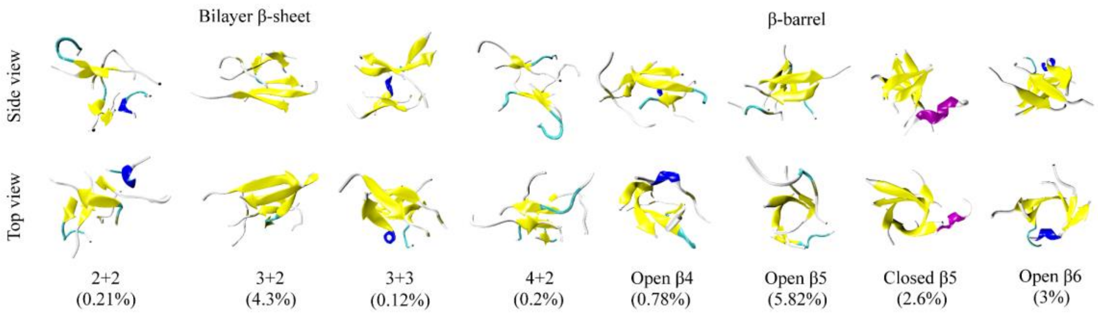

Figure S7. Representative TTR (105-115) conformations of bilayer β-sheet and β-barrel from the cluster analysis of MD simulation for the six-peptides system. Side view and top view were shown. The size of bilayer β-sheet were represented *i+j*, where *i* and *j* showed the *i* and *j* stranded β-sheet in each layer, respectively. the n of βn represented n stranded β-barrel, and the type of β-barrel was classed by open and closed.

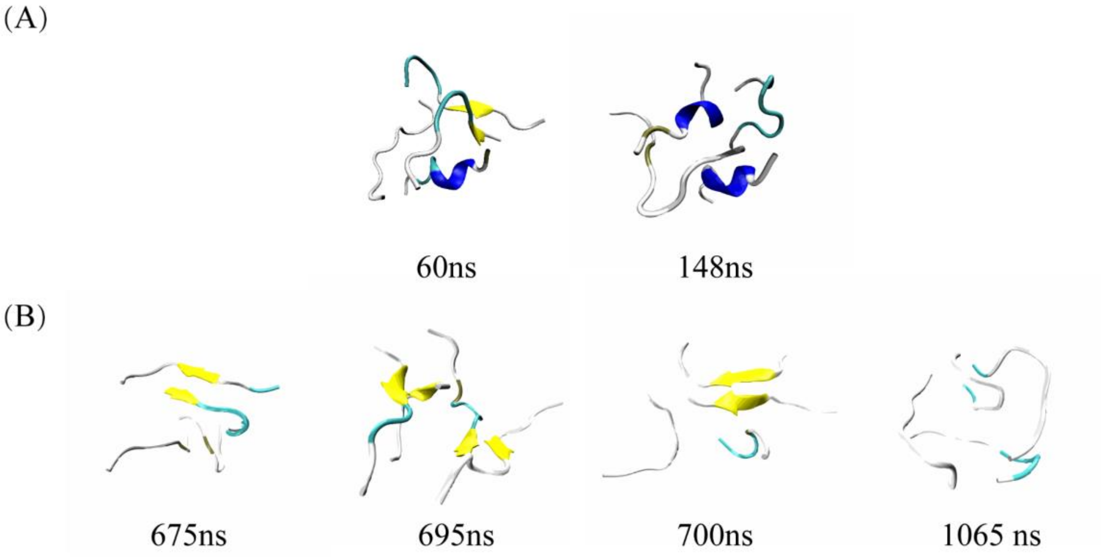

Figure S8. The representative structures changes of four-peptides were presented with the time evolution. (A) The process by which a pair of β-sheet became random coil. (B) Two pairs of dimers with β-sheet contents were generated and all peptides subsequently converted to a random coil state.
